# Pulmonary extra‐nodal mucosa‐associated lymphoid tissue (MALT) lymphoma: A rare cause of persistent lung consolidation

**DOI:** 10.1002/rcr2.1197

**Published:** 2023-07-25

**Authors:** Lily Kou, Nai‐Chien Huan, Larry Ellee Nyanti, Jiunn Sheng Chin, Nor Bahiyyah Mohamad, Hema Yamini Ramarmuty

**Affiliations:** ^1^ Department of Medicine Keningau Hospital Keningau Malaysia; ^2^ Department of Respiratory Medicine Queen Elizabeth Hospital Kota Kinabalu Malaysia; ^3^ Medical Department, Faculty of Medicine and Health Sciences Universiti Malaysia Sabah Kota Kinabalu Malaysia; ^4^ Department of Pathology Queen Elizabeth Hospital Kota Kinabalu Malaysia

**Keywords:** lung consolidation, lymphoma, pulmonary mucosa‐associated lymphoid tissue

## Abstract

Pulmonary extra‐nodal marginal zone B‐cell lymphoma, also known as extra‐nodal mucosa‐associated lymphoid tissue (MALT) lymphoma, is rare among all non‐Hodgkin lymphomas and generally among all pulmonary malignancies. We present a 46‐year‐old lady with persistent right lower lung consolidation despite earlier treatment efforts with intravenous antibiotics for community acquired pneumonia. Apart from initial presentation with a short 3‐day history of fever, cough and shortness of breath, she had remained largely asymptomatic throughout the follow‐up period. Flexible bronchoscopy done ruled out infectious aetiologies but transbronchial lung biopsies showed atypical lymphocytes. A computed tomography guided core biopsy of her right lung consolidation was subsequently performed, confirming a diagnosis of pulmonary MALT lymphoma. She was promptly referred to the haematology team for further management and commencement of chemotherapy. Pulmonary MALT lymphoma, albeit uncommon and often follows a relatively indolent cause, should be considered as a differential diagnosis among patients with persistent lung consolidation.

## Graphical abstract

Primary pulmonary lymphoma is a rare clinical entity, accounting for less than 1% of all lymphomas. Pulmonary MALT lymphoma typically follows an indolent course, and nearly half of patients are asymptomatic at diagnosis. A high index of suspicion is therefore required to confirm the diagnosis. Herein, a case of pulmonary MALT lymphoma in a middle‐aged lady with persistent right lung consolidation is described.

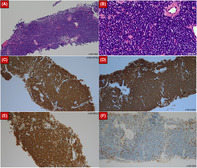

## INTRODUCTION

Primary pulmonary lymphoma (PPL) is a rare clinical entity, accounting for less than 1% of all lymphomas and only 3%–4% of all extra‐nodal lymphomas.^1^ Pulmonary extra‐nodal marginal zone B‐cell lymphoma (MZL), also known as extra‐nodal mucosa‐associated lymphoid tissue (MALT lymphoma), is the most common form of PPL despite accounting for less than 1% of all pulmonary malignancies.^1^ Pulmonary MALT lymphoma typically follows an indolent course, and nearly half of the patients are asymptomatic at diagnosis.^1^ A high index of suspicion is therefore required to confirm the diagnosis. Herein, a case of pulmonary MALT lymphoma in a middle‐aged lady with persistent right lung consolidation is described.

## CASE REPORT

**FIGURE 1:**
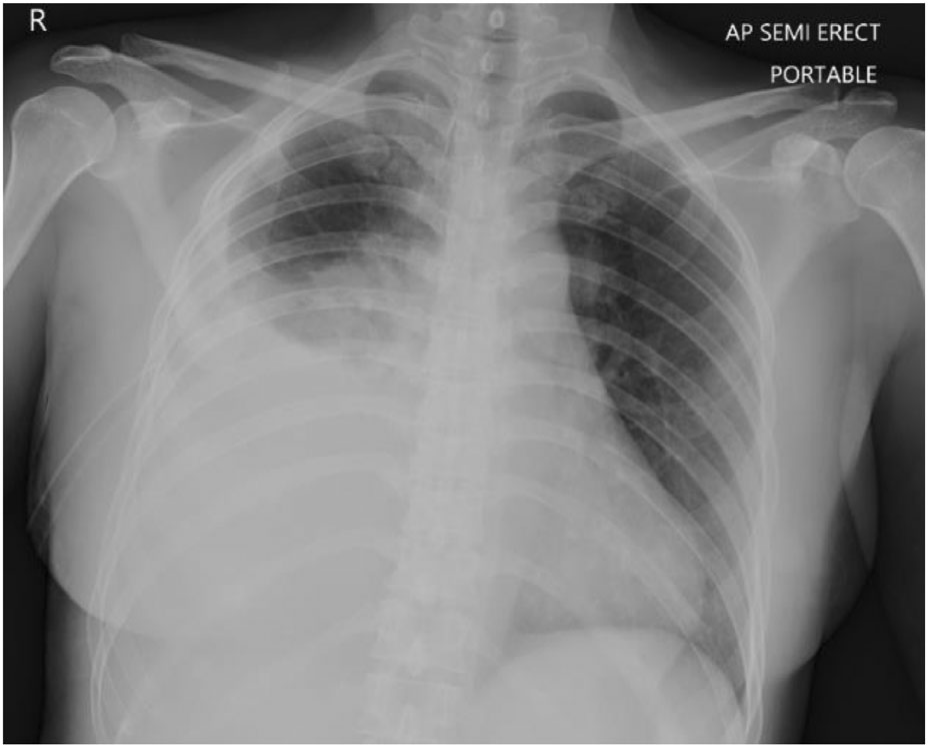
Chest radiograph during initial presentation showing a right lower zone opacity due to lung consolidation and surrounding pleural effusion.

A 46‐year‐old lady, non‐smoker, presented with 3 days of non‐productive cough, dyspnoea, right pleuritic chest pain and fever. Upon admission, she had a blood pressure of 162/99 mmHg, pulse rate of 126 beats/min, oxygen saturation of 95% under room air and temperature of 39.4°C. Examination of the thorax revealed stony dullness and reduced air entry at the right lower zone. Blood investigations revealed leucocytosis at 22.9 × 10^9^ (neutrophils predominant) and elevated C‐reactive protein at 280 mg/L. Chest radiograph showed a right lower zone consolidation with a blunted costophrenic angle (Figure 1), while bedside thoracic ultrasound demonstrated an anechoic right pleural effusion. Thoracocentesis confirmed an exudative effusion with pleural fluid lactate dehydrogenase of 905 IU/L, pleural fluid protein of 42 g/dL (pleural/serum protein ratio 0.6), and pleural fluid glucose of 28 g/L. Pleural fluid bacterial and tuberculous cultures, gram staining, acid‐fast bacilli staining and cytology were all negative, while pleural fluid adenosine deaminase was not elevated at 20.2 U/L.

**FIGURE 2:**
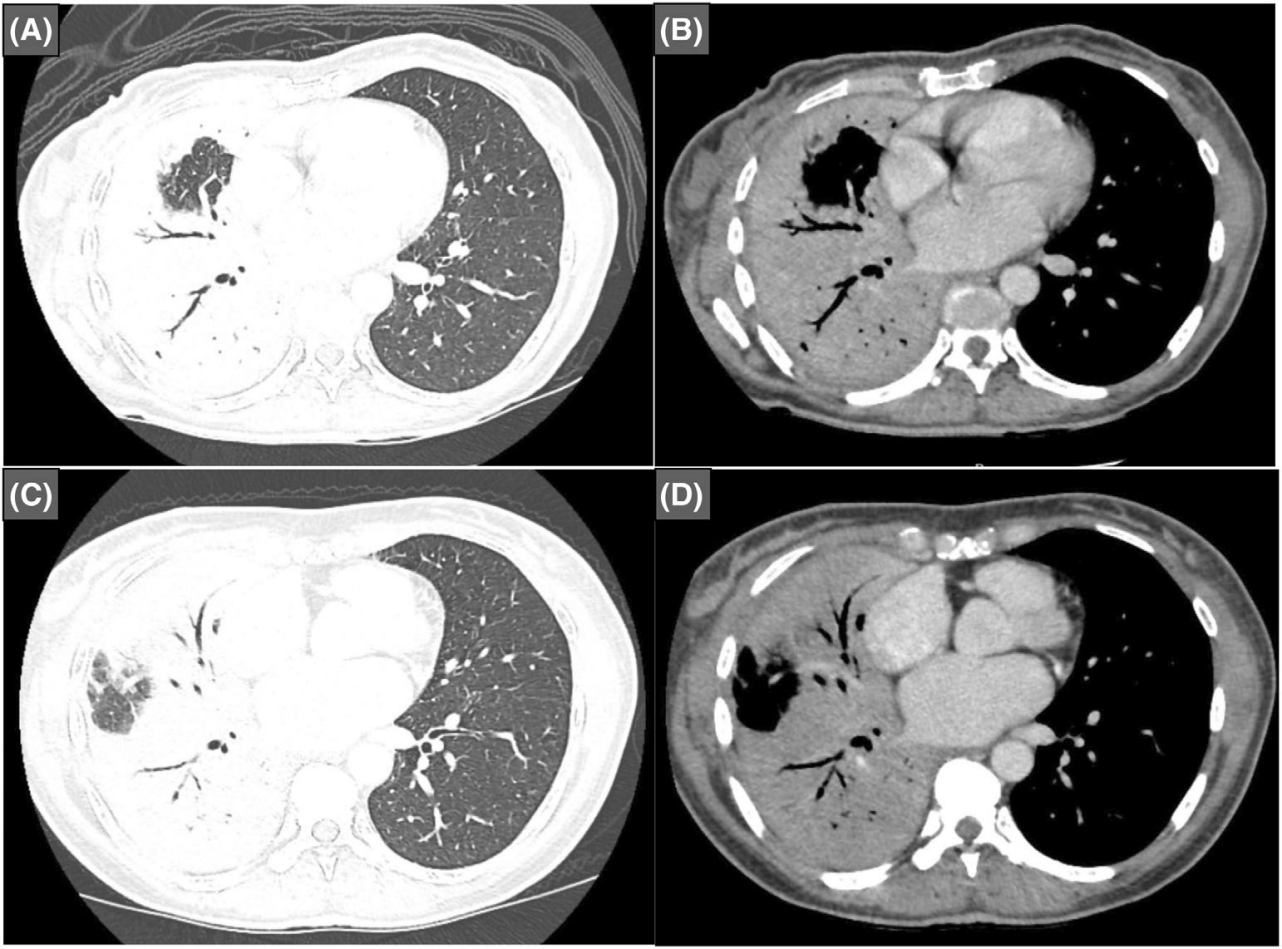
(A, B) Computed tomography (CT) of the thorax done approximately 2 months after initial presentation demonstrating persistent right lung consolidation. (C, D) Repeat CT thorax 6 months after the initial CT again revealing a persistent right lung consolidation, showing no response towards anti‐tuberculous medications.

Intravenous ampicillin‐sulbactam was commenced and a chest drain was inserted to treat a presumed community‐acquired pneumonia with right parapneumonic effusion; the patient made good recovery and was discharged well. During her outpatient follow‐up clinic 2 months later, she reported no respiratory or constitutional symptoms, but a repeated chest radiograph and computed tomography (CT) of the thorax (Figure 2A, B) revealed a persistent consolidative opacity in the right lower lobe. The treating team decided to empirically treat her for presumed pulmonary tuberculosis in view of persistent lung consolidation and a remote history of household tuberculosis contact. Nevertheless, another repeated CT thorax 6 months later showed an unchanged lung consolidation (Figure 2C, D) despite the completion of anti‐tuberculous medications for a total of 6 months. A bronchoscopy was therefore performed and bronchial washings sent for mycobacterial, fungal and bacterial cultures were negative. Transbronchial lung biopsy demonstrated clusters of atypical lymphoid cells, however, further immunohistochemical analysis could not be performed due to insufficient biopsy volume.

**FIGURE 3:**
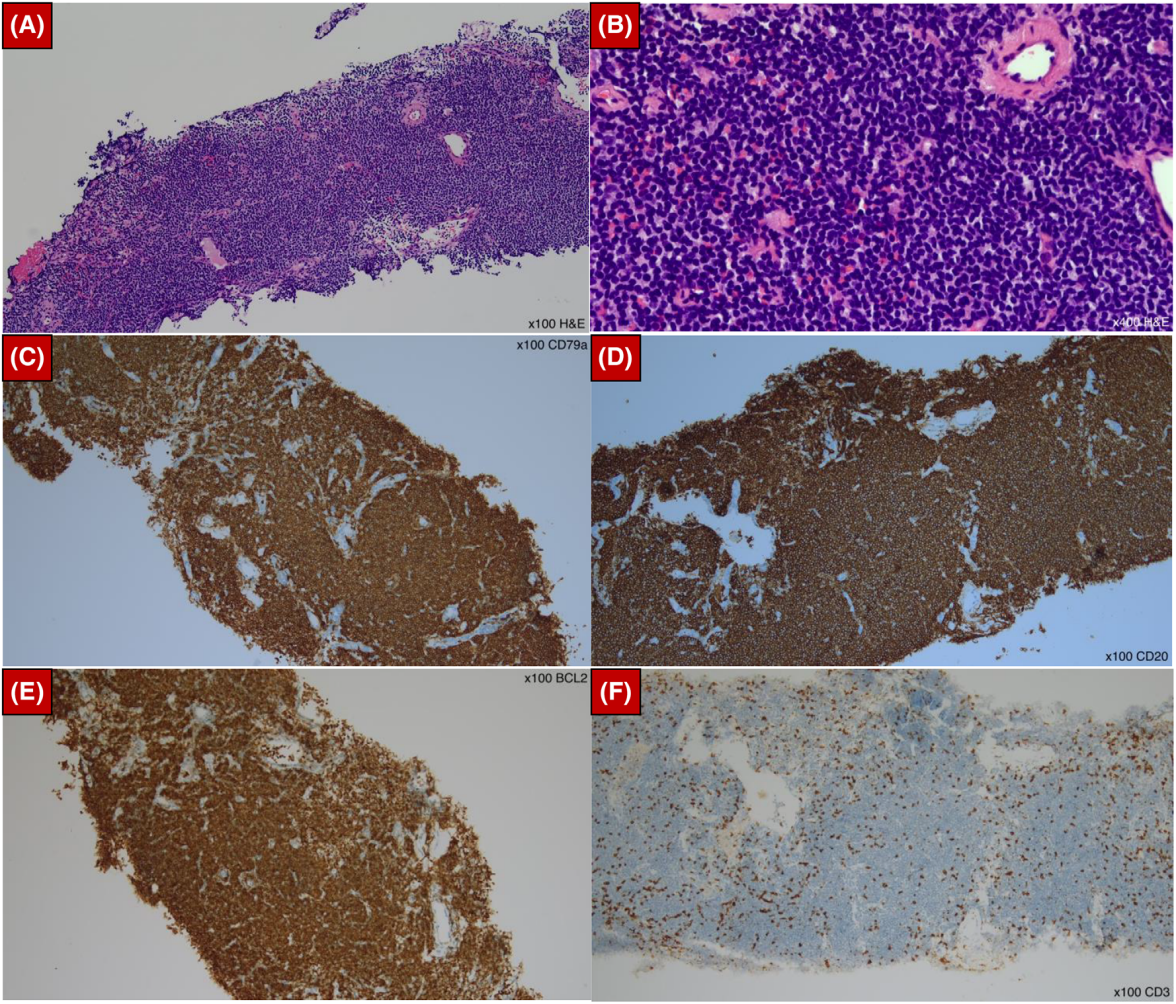
CT guided biopsy of lung consolidation confirmed a diagnosis of pulmonary MALT lymphoma. (A) Lung tissue section at 100 times magnification showing sheets of atypical lymphoid cells with scattered hyalinized vessels. (B) Further magnification at 400 times of the same segment of lung tissue showing atypical lymphoid cells that are mostly small sized, with irregular nuclei, dispersed chromatin, and inconspicuous nucleoli. Occasional interspersed larger cells are seen as well. (C–E) The cells were diffusely positive for B cell markers of CD79a (C), CD20 (D) and BCL2 (E). (F) CD3 and CD5 markers highlight the non‐neoplastic T cells in the background, clearly showing that the tumour cells were negative for both CD3 and CD5.

Subsequent CT‐guided percutaneous biopsy demonstrated interstitial expansion by atypical lymphoid cells. Immunohistochemistry was positive for CD20, CD79s and BCL‐2 and negative for CD3, CD5, CD10, CD23, BCL6 and Cyclin D1. Immunostaining for kappa and lambda showed findings suggestive of weak kappa expression (Figure 3A–E). The overall morphological and immunohistochemical features were diagnostic for B‐cell non‐Hodgkin lymphoma of extra‐nodal MALT origin. Further staging revealed no thoracic, abdominal or pelvic lymphadenopathy, but oesophagogastroduodenoscopy (ODGS) biopsies confirmed gastric MALT lymphoma as well. Further evaluation via trephine biopsy showed normocellular marrow with no evidence of lymphomatous infiltration, and she was commenced on chemotherapy with R‐CHOP regime (cyclophosphamide, doxorubicin, vincristine and prednisolone plus rituximab).

## DISCUSSION

Pulmonary consolidation occurs when normal air‐filled alveoli of the lung are replaced by pathological products such as fluid, blood, pus, interstitial fluid and gastric contents. Abnormal proliferation of clonal marginal zone lymphocytes is responsible for lung consolidation among patients with pulmonary MALT lymphoma. Guidelines recommend repeating imaging 6 weeks after the initial pulmonary infection to look for resolving consolidation.^2^ In the absence of positive cultures or evidence of ongoing infection, a persistent lung consolidation should prompt the clinician to consider non‐infectious aetiologies including inflammatory diseases, autoimmune conditions and malignancies.

Pulmonary MALT lymphoma is a rare disease with a median age of diagnosis at 50–60 years.^3^ It can occur secondary to chronic antigen stimulation by either infectious pathogens or autoimmune mechanisms leading to uncontrolled lymphocyte proliferation.^4^ Therefore, patients with pulmonary MALT should be screened for infectious or autoimmune conditions such as rheumatoid arthritis, Sjogren's syndrome, collagen vascular diseases, acquired immunodeficiency syndromes and *Helicobacter pylori* infections. We performed screening for both infectious and autoimmune conditions in our patient but all were unremarkable.

Clinical manifestations of pulmonary MALT lymphoma are non‐specific, including cough, haemoptysis, chest pain and dyspnoea. The majority of patients are asymptomatic, as in our case.^1^ The duration between initial symptoms and diagnosis may span several months or even years, highlighting the need for a high degree of clinical suspicion.^5^ Radiologically, they manifest as features which overlap with infectious pathology such as consolidation, air bronchograms, masses or nodules,^6^ making diagnosis difficult. Based on the pattern of parenchymal abnormalities, pulmonary MALT lymphoma is classified into four types: pneumonia‐like consolidation pattern, nodular or mass pattern, ground‐glass opacity pattern and diffuse interstitial lung disease pattern.^7^ Bronchiectasis within lung consolidation with bulging of interlobar fissures are more frequently encountered in patients with MALT lymphoma than those with lobar pneumonia.

A definitive diagnosis of pulmonary MALT lymphoma can only be achieved following histopathological analysis of biopsy specimens obtained from minimally invasive procedures such as transbronchial biopsies and/or radiologically guided samplings. Besides, due to the possibility of multi‐lobar lesions or disseminated multisystem disease, a single minimally invasive procedure might not be sufficient to clinch a full diagnosis.^5^ Our patient had to undergo a few procedures: namely bronchoscopy, CT guided lung biopsy and later OGDS before a full diagnosis was made. This was further compounded by the initial decision of the treating team to empirically treat her for presumed tuberculosis, which is a common practice in many tuberculosis‐endemic regions but retrospectively was not a right decision.

While most cases of pulmonary MALT lymphoma are localized and indolent, it can potentially transform into high‐grade B‐cell lymphoma if left untreated. Earlier studies by Borie et al. reported that the estimated 5 and 10‐year survival rates were at 90% and 72%, respectively.^8^ The optimal treatment strategy for pulmonary MALT lymphoma remains undefined, especially among those who are asymptomatic at diagnosis. Troch et al. suggested watchful waiting for patients who are asymptomatic at diagnosis.^9^ In contrast, Ahmed et al. in their report involving 22 patients with MALT lymphoma demonstrated that four out of six patients who were initially observed ultimately require chemotherapy in view of disease progression.^10^ Our patient was treated with chemotherapy despite being relatively asymptomatic due to concurrent involvement of oesophagus and due to fear of further disease progression.

In conclusion, a high index of suspicion and prompt decision for biopsy (whilst avoiding unnecessary treatment for presumed tuberculosis in absence of strong clinical and radiological evidence) in a patient with non‐resolving lung consolidation are required to confirm the diagnosis of pulmonary MALT lymphoma.

## CONFLICT OF INTEREST STATEMENT

None declared.

## ETHICS STATEMENT

The authors declare that appropriate written informed consent was obtained for the publication of this manuscript and accompanying images.

## Data Availability

The data that support the findings of this study are available from the corresponding author upon reasonable request.
